# Modified protrusive wax record in recording condylar path angle and ethnic variations

**Published:** 2017-05-24

**Authors:** Ammar Musawi, Yusnidar Tajul Ariffin

**Affiliations:** 1 Missouri School of Dentistry & Oral Health, A.T. Still University, 800 W. Jefferson St., Kirksville, Missouri, United States; 2 University of Malaya, Kuala Lumpur, Malaysia

**Keywords:** condylar angle, ethnic variation, modified protrusive wax method

## Abstract

**Background**: The Condylar Path Angle (CPA) is an important measurement that is used to program articulators used in dental treatment. The purpose of the current study was to investigate the CPA in Malay subjects, to compare the measurements with average values, 25˚-35˚ Camper’s (based on Caucasian studies), and to compare the right and left CPAs.

**Methods**: Thirty subjects aged 21-23 years were recruited. A wash technique impression was made, casts were poured, and face-bow transfers were taken. The casts were mounted to their centric position on a semi-adjustable articulator. Protrusive guides were constructed to allow the mandible to be protruded for 5 mm, and then the angles were measured using the protrusive record method.

**Results**: The right CPA was within the normal range for 43% (13/30) of participants and out of the normal range for 57% (17/30). The left CPA was within the normal range for 33% (10/30) of participants and out of the normal range for 67% (19/30). There was no statistically significant difference between the left and right CPAs (*p* = 0.72), but there was a strong linear relationship between left and right CPAs (*p* = 0.001). **Conclusions**: Results of the current study indicated Malay subjects had measurable variations in the CPA, suggesting this population has an ethnic variation in the CPA.

**Relevance for patients**: To improve the quality of patient care, the CPA should be considered when constructing fixed/removable prostheses that use semi-adjustable articulators, and clinicians should not rely on the set average values that are pre-set on articulators.

## 1. Introduction

Articulators are frequently used during prosthodontic treatment. Semi-adjustable articulators allow adjustment of the condylar path angle (CPA), average value advised for the setting is 30˚ Campers, based on previous research [1], and Bennett movement with a fixed intercondylar width of 110 mm [2]. More recent semi-adjustable articulators offer different intercondylar width settings. Quick and accurate programming of a semi-adjustable articulator to simulate functional and para-functional movements is necessary for the articulator to work efficiently. Inaccurate programming will lead to inefficient treatment planning and inappropriate treatment [3].

There are 3 different methods for measuring CPA. One method uses intra-oral wax records and then the angle is calculated on the articulator [4,5]. Another method records the CPA on a card using a face bow; correct adaptation is facilitated by an intra-oral bearing device to adjust the articulator setting, and the angle of the path is obtained by measuring the tangent of the functional portion of the tracing [6,7]. The final method involves the use of mandibular tracking devices [8].

Research suggests that there are ethnic differences in the CPA. In a 1985 study of Chinese students [9], mean Sagittal condylar guidance angles was 11.30 for the right and 11.70 for the left compared to 25.30 for the right and 24.90 for the left for the Caucasian population, ethnic differences were found between the students and a previously published sample from a Caucasian population; dental articulators and measurement of the CPA were used. A significant difference was also found between the sagittal condylar guidance angles and the angle of the occlusal plane to the Frankfort plane as measured by a prosthetic technique when comparing Nigerian participants with Caucasians, which suggested that different ethnic groups should be examined to see if anatomical differences exist that may invalidate the use of articulators designed for a Caucasian population [9]. In a study of Cantonese patients [10], anatomical differences were found for this ethnic group, such as variations in the orientation of the Frankfort plane and the occlusal plane.

Given these previous studies suggesting ethnic differences in CPA, the purpose of the current study was to investigate the CPA in Malay subjects, to compare the measurements with average values, and to compare the right and left CPAs.

## 2. Methods

Participants aged between 21 and 23 years were recruited for the current study from a dental university in Malaysia. Selection criteria included this age range to ensure full growth of the temporomandibular joint (TMJ). Potential participants had to be part of the Malay ethnic group with pure Malay parents and grandparents. They also had to be fully dentate with no history of dental extractions and have no orthodontic history or gross restorations and bridges. The TMJ area had to be healthy with no history of trauma or disease. Finally, the participant had to be able to protrude the mandible for a minimum of 5 mm. Potential participants with class II orthodontic classification with deep bite were excluded from the study. The local ethics committee approved all study procedures, and participants signed informed consent forms.

Impressions for maxillary and mandibular arches were made with putty-type silicone impression material (EXAFLEX, GC America, Alsip, IL) according to the manufacturer’s instructions. During this process, the impression tray was moved back and forth and sideways to create space for the wash impression using the regular body silicone impression material of the next step. After the putty material was set, the tray was removed from the participant’s mouth. Any excess material or undercut areas in the impression were removed to allow easy reseating of the impression inside the participant’s mouth.

The regular body silicone impression material, hydrophilic vinyl polysiloxane regular type (EXAFLEX), was mixed, put into the putty impression, and then re-seated back in the participant’s mouth. The impression was considered acceptable if it recorded all the teeth and was clear of voids or deformation. The impression was washed, disinfected, and then sent to the laboratory to be poured.

The impression was poured with type IV dental die stone according to manufacturer’s mixing ratios (24 mL/100 g). After the stone was set, the impression was removed from the cast and allowed to dry by air for 48 hours to produce hard surface. Casts were then labeled and numbered.

The KaVo facebow instrument (Biberach, Germany) was used to transfer the 3-dimensional relationship between the participant’s maxillary arch and mandibular condyles. This relationship was recorded and transferred during mounting of the maxillary cast to the semi-adjustable articulator (KaVo PROTARevo 9 ArCon). Centric occlusion was recorded. The material used for the bite registration record was silicone bite registration material (Exabite II NDS, GC America, Alsip, IL), and the mandibular cast was mounted accordingly.

The condylar and the Bennett angles were set to default values (30° Camper’s for the condylar angle and 15˚ Bennett). The facebow record was attached to the transfer jig on the articulator, and the maxillary cast was seated properly on the record. Adequate space between the base of the cast and the mounting plate was verified so there would be enough space for the mounting of the casts on the articulator with plaster of Paris.

## 3. Protrusive Guide Construction

**Figure 1.**
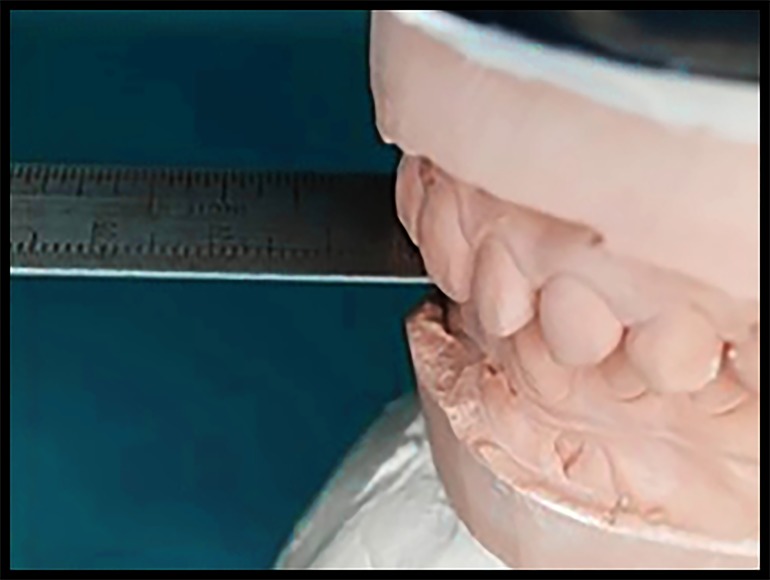
Measuring the horizontal overlap on the cast using a ruler

**Figure 2.**
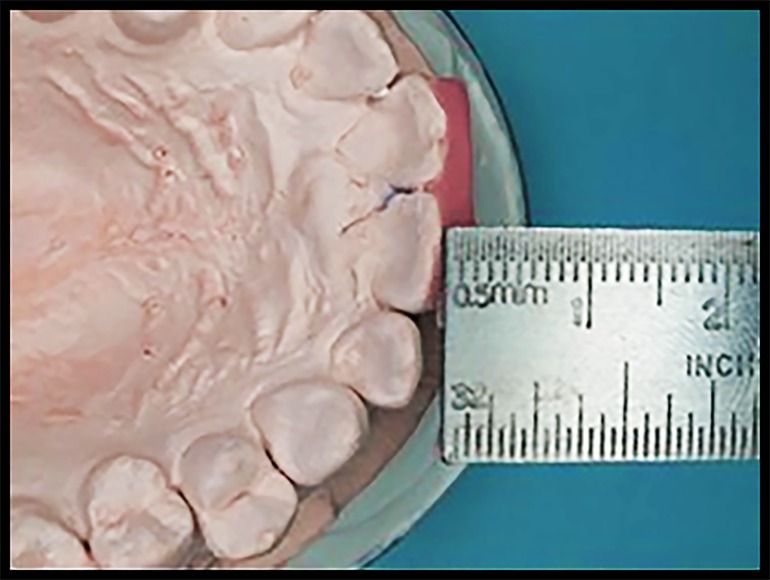
Measuring the thickness of the adapted resin on the anterior teeth

The protrusive guide was prepared on the maxillary cast by using light-activated acrylic resin that was cut into rectangular-shaped plates (1.5 cm × 1.0 cm) and light cured. The distance between the labial surface of the mandibular central incisors and the labial surface of the maxillary central incisors was measured (Figures 1 and 2) to ensure that the participant always protruded for 5 mm exactly. After that, the light-cured resin was adapted to the labial surface of the maxillary anterior teeth, and the thickness of the material was determined using the following formula: amount of horizontal overlap + thickness of the resin material = 5 mm protrusion distance.

**Figure 3.**
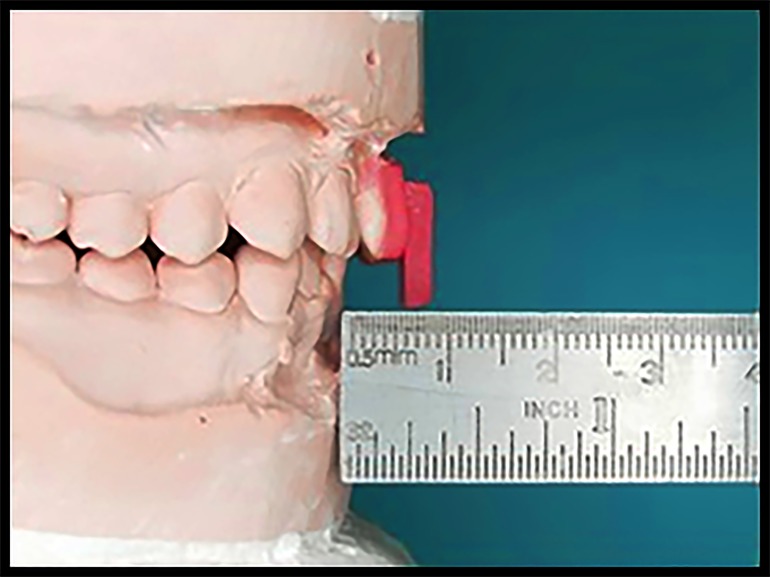
Verifying the 5 mm distance between the protrusive guide and the labial surface of the lower anterior teeth

The maxillary cast with the adapted material was light cured, and the thickness of the material was checked after 24 hours for any changes of the acrylic resin material from polymerization shrinkage. Any such changes to the thickness were adjusted accordingly. The previously prepared rectangular plates were then attached to the outer surface of the adapted material on the central incisors (Figure 3).

## 4. Protrusive Record

**Figure 4.**
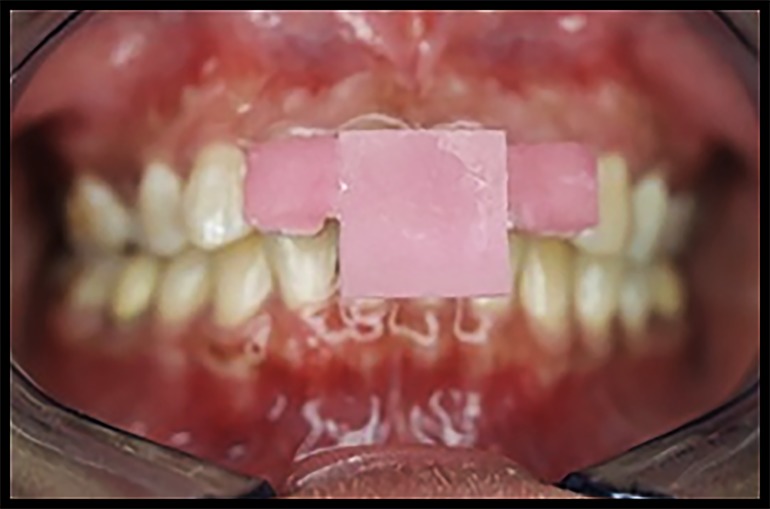
Protrusive guide cemented on the maxillary anterior teeth

Prior to recording the protrusive records, the protrusive guide was cemented on the participant’s maxillary incisors using temporary cement and held in place until the cement set (Figure 4).

**Figure 5.**
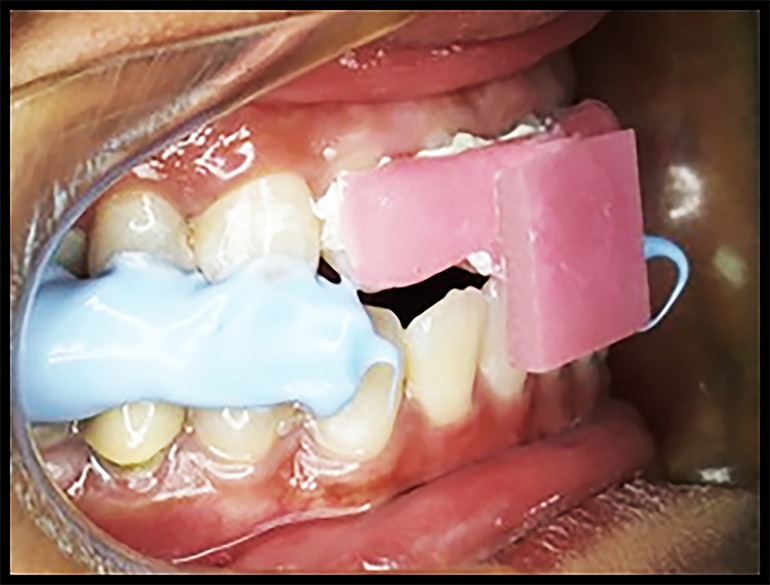
Registration of the protrusive position with silicone bite registration material

Participants were trained on how to perform the protrusive movement by the operator. Each participant was required to slowly protrude from the centric position until the mandibular incisors gently touched the plate of the protrusive guide. The participant held that position and then raised a hand to signal that the silicone bite registration material could be injected between the occlusal surfaces of the premolars and molars (Figure 5).

After allowing the registration to fully harden, the record was removed from the participant’s mouth, washed with tap water, and disinfected. Each pair of records were marked with the same mark and transferred to the mounted casts for programming of the facebow-mounted models. This procedure was repeated 3 times for each participant, mean value was recorded.

**Figure 6.**
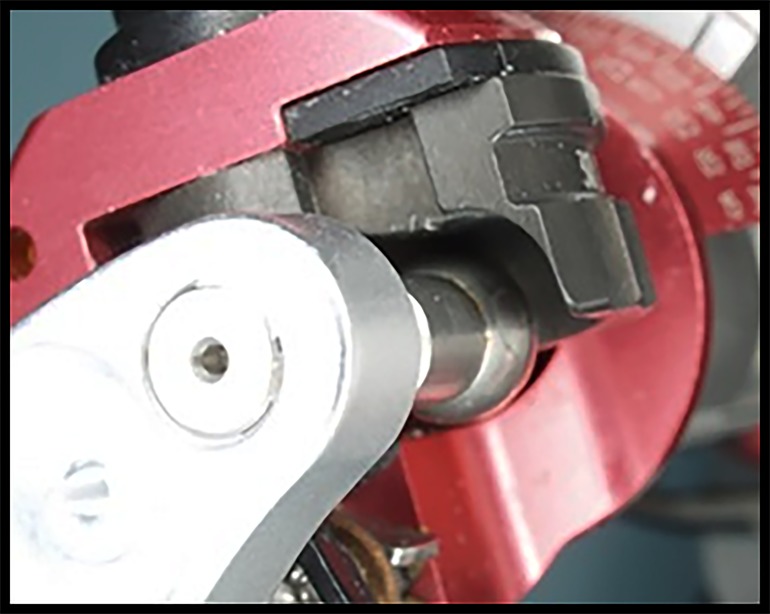
Condyle of the articulator not in contact with the eminence

After releasing the condylar locks on the articulator, the protrusive records were placed on the models and fully seated (observe the condylar head replica off the eminence slope of the articulator) (Figure 6). If the condylar head was in contact with the eminence, it was adjusted to avoid interference with the complete seating of the model in the protrusive record. Once the models were completely seated, the condylar angle pathway locks were released so that the condylar angle could be adjusted without resistance.

**Figure 7.**
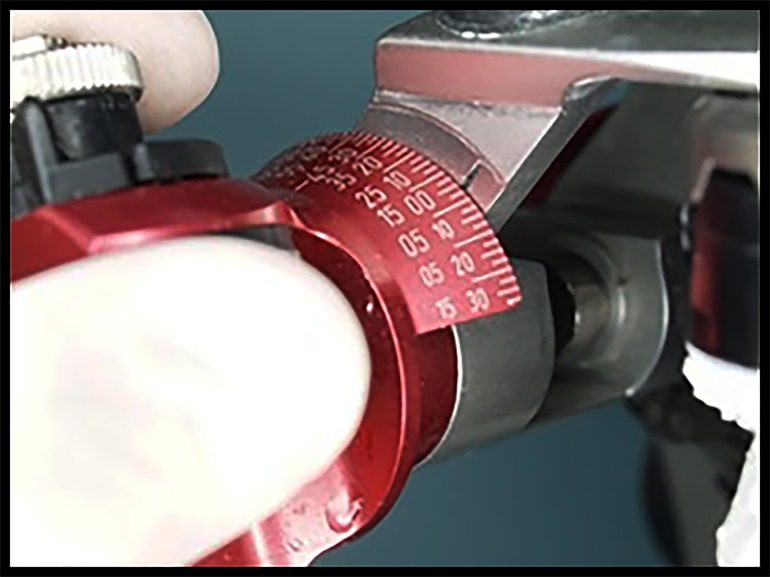
Adjusting the condylar sagittal path angle

With the casts fully seated in the protrusive position, the eminence slope of the articulator was rotated until contact was made with the condylar head replica of the articulator (Figure 7). The condylar angle pathway locks were re-engaged, and the condylar angle was noted and the measurement recorded.

## 5. Statistical Analysis

The measurements were repeated 3 times to verify the reliability of the measuring technique. Cronbach’s α was used to test the reliability and was 99% for right side measurements and 95% for left side measurements. The means and standard deviations were calculated for all variables measured and were used for subsequent analyses. A paired *t* test was used to compare the right and left CPA of participants. A Pearson test was used to determine if there was any linear relationship between the right and left sagittal CPAs. SPSS statistical software version 12.0 (SPSS Inc., Chicago, IL) was used for all analyses. The significance level was set *p* ≤ 0.05.

## 6. Results

Thirty dental students of the Malay ethnic group completed the current study. Their ages ranged from 21 to 23 years.

**Figure 8.**
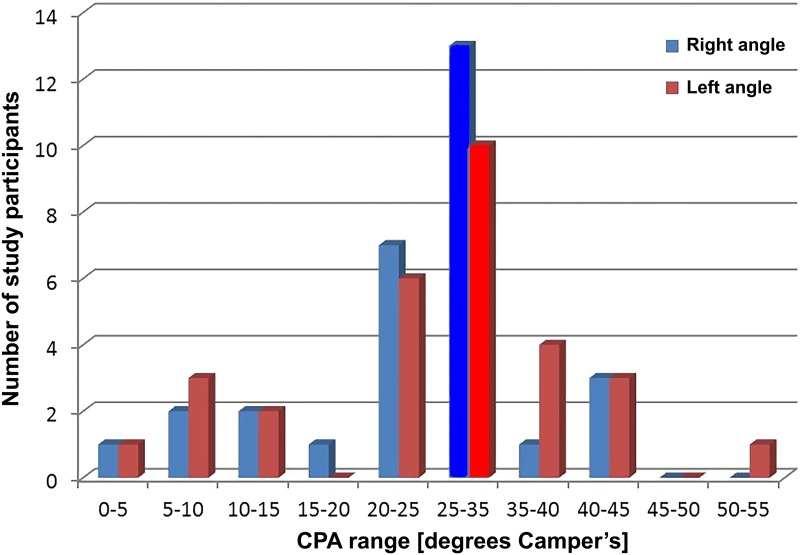
Distribution of the condylar angle recordings for the right side and left side

The mean (SD) was 26.4° (10.1) for the right CPA and 26.7° (12.0) for the left CPA, and the mean difference was –0.35 (95% confidence interval, 2.3-1.6). Most of the participants did not fall within the normal range suggested for pre-setting the articulators (30°). For the right CPA, only 13 (43%) of 30 participants were within the normal range; for the left CPA, only 10 (33%) of 30 were within the normal range (Figure 8).

There was no statistically significant difference between the right and left CPAs (*t*_29_ = −0.36, *p* = 0.72), but there was a strong linear relationship between the right and left CPAs (Pearson *r* = 0.90, *p* = 0.001).

## 7. Discussion

Few dentists make full use of semi-adjustable articulators. Because they can be adjusted to simulate jaw movement more accurately, their use can minimize chairside corrections of occlusal discrepancies during prosthodontic treatment, whether fixed or removable.

The protrusive record method for measuring CPA is the most feasible and least complicated method to use in clinical practice because it is not expensive and does not require specialized devices or machinery [11]. As such, every prosthodontist, general practitioner, or dental student can use this technique to record the CPA. However, the main concern regarding the wax protrusive method is the difficulty of having the patient repeat the same protrusive position. The existing literature has repeatedly suggested this method is unreliable, unrepeatable, invalid, and arbitrary. As long ago as 1964, Carlsson and Arstrand [12] suggested that check bite condylar registration of condylar path inclination in patients with complete denture should be eliminated from dental education. In 1984, Ecker et al[13] stated that wax protrusive records did not provide consistent readings, but the discrepancies did not contraindicate their use with semi-adjustable instruments. To address the shortcomings of this technique and improve its reliability, modifications were made to the wax protrusive method in the current study. For instance, we used silicone bite registration material instead of wax because it is highly accurate and easier to use and has good dimensional stability [14]. We also used a protrusive guide, which allowed the participant to protrude the mandible in the same position each time the record was made [15,16].

In the current study, the CPA’s of the Malay ethnic group was investigated and compared to the average values, it was found that the right CPA was within the normal range for 43% of participants, and the left CPA was within the normal range for 33% of participants. The CPAs for both sides were out of the normal range for the majority of our participants. These results support the findings of Melkers [3]. In that study [3], 46% of measurements in 54 participants did not fall within average values, and there was a wide distribution of findings. Results of the current study also supported those of Chow et al [10], who examined 32 participants of a Cantonese ethnic group and found significant variation in Camper’s angle. In another study, Fletcher [9] suggested that articulators should not be used with patients in the Chinese Singaporean ethnic group of that study because of variations in CPA when compared with a Caucasian population. When measuring the CPA of the Malay ethic group of the current study, our results also suggested an ethnic variation for the CPA.

The current study also found a strong linear relationship between the right and left CPAs. This result may be explained by the specifics of the TMJ because the right and left TMJs do not act as separate joints and move in coordination with one another. Therefore, a strong linear relationship would be expected. Sample size of the study is considered to be a limitation; however, the sample does reflect an issue that needs to be investigated further.

## 8. Practical Implications

Based on the results of the current study and previous studies, it is recommended that practitioners do not rely on the average value settings of articulators when doing complicated prosthodontic treatments on patients. As demonstrated, there are ethnic variations in the CPA measurements in addition to already existing variations in the Caucasian population.

## 9. Conclusion

The results of the current study suggested that the Malay ethnic group studied had an ethnic variation in relation to the CPA. The right and left CPAs were comparable to each other, and they had a strong linear relationship. For dental practitioners performing prosthodontic treatments, this ethnic variation should be considered when comparing the average values of CPA measurements.
